# Is the Best Evidence Good Enough: Quality Assessment and Factor Analysis of Meta-Analyses on Depression

**DOI:** 10.1371/journal.pone.0157808

**Published:** 2016-06-23

**Authors:** Yingbo Zhu, Lin Fan, Han Zhang, Meijuan Wang, Xinchun Mei, Jiaojiao Hou, Zhongyong Shi, Yu Shuai, Yuan Shen

**Affiliations:** 1 Department of Psychiatry, Shanghai Tenth People’s Hospital, Tongji University, School of Medicine, Shanghai, China; 2 Department of Orthopedics, Shanghai Tenth People’s Hospital, Tongji University, School of Medicine, Shanghai, China; 3 Department of Hepatic Surgery, Eastern Hepatobiliary Surgery Hospital, Second Military Medical University, Shanghai, China; Toronto Western Hospital, CANADA

## Abstract

**Background:**

The quality of meta-analyses (MAs) on depression remains uninvestigated.

**Objective:**

To assess the overall reporting and methodological qualities of MAs on depression and to explore potential factors influencing both qualities.

**Methods:**

MAs investigating epidemiology and interventions for depression published in the most recent year (2014–2015) were selected from PubMed, EMBASE, PsycINFO and Cochrane Library. The characteristics of the included studies were collected and the total and per-item quality scores of the included studies were calculated based on the two checklists. Univariate and multivariate linear regression analyses were used to explore the potential factors influencing the quality of the articles.

**Results:**

A total of 217 MAs from 74 peer-reviewed journals were included. The mean score of Preferred Reporting Items for Systematic Reviews and Meta-Analyses (PRISMA) was 23.0 of 27 and mean score of Assessment of Multiple Systematic Reviews (AMSTAR) was 8.3 of 11. Items assessing registration and protocol (14.2%, 37/217) in PRISMA and item requiring a full list of included and excluded studies (16.1%, 40/217) in AMSTAR had poorer adherences than other items. The MAs that included only RCTs, pre-registered, had five more authors or authors from Cochrane groups and the MAs found negative results had better reporting and methodological qualities.

**Conclusions:**

The reporting and methodological qualities of MAs on depression remained to be improved. Design of included studies, characteristics of authors and pre-registration in PROSPERO database are important factors influencing quality of MAs in the field of depression.

## Introduction

Depression is a common mental disorder with high prevalence and mortality [1]. It will be ranked second among the top 10 leading causes of the global burden of diseases in 2020 [2]. Over the last decades, publications in the field of depression increased dramatically, as demonstrated by a crude search using MeSH word “depression” and article type filter “meta-analysis” on PubMed showing a 4.4-fold increase in number of meta-analyses (MAs) published in the 2005–2015 peroid compared with the 1995–2005 peroid (2206 vs 502). MAs provide a comprehensive and objective appraisal of evidence were recognized as higher-level evidence than other research types. In the age of evidence-based medicine, MAs are developing rapidly with the number of publication increasing annually. MAs can help to resolve conflicting evidence, identify gaps, and provide valuable conclusions; thus, they are increasingly important in assisting clinicians to make evidence-based decisions [3,4]. However, it is recognized that poor reporting [5,6] and methodological quality may introduce bias and impair the reliability of conclusions although rigorous methodology is a feature of MAs [7]. As conclusions from MAs are often important references for decision-making, poor reporting and methodological quality of MAs may interfere the delivery of appropriate information to clinicians and policy makers.

Several standards were produced in an attempt to improve the research quality of MAs in recent years. Preferred Reporting Items for Systematic Reviews and Meta-Analyses (PRISMA) is the updated version of Quality of Reporting of Meta-Analyses (QUOROM), which was the first guideline for assessing reporting quality of MAs [8,9]. PRISMA checklist is a standard frame developed to check whether authors have reported results adequately and transparently. It has been widely utilized as a standard to evaluate the reporting quality of MAs since it was released in 2009 [8]. Meanwhile, several standards have been developed to assess methodological quality of MAs [10], among which the Assessment of Multiple Systematic Reviews (AMSTAR) is a well-acknowledged tool for evaluating the methodological quality of MAs [11]. It is more prescriptive as an appraisal measurement rather than a reporting guideline. AMSTAR has been proved to have good agreement, reliability, construct validity and feasibility in identifying scientific flaws and extent of bias of MAs [11,12].

Research in depression presents particular challenges for providing high-quality evidence. Psychiatric interventions are difficult to standardize due to large differences between both individual patients and physicians, particularly for psychological therapies. Moreover, co-interventions are very common in this field, e.g., medication combined with psychotherapy and medication combined with physical therapy. Thus, the interaction between therapies may lead to confounding results. In this regard, psychiatric practice is less likely to be based on evidence when compared with clinical medicine [13]. A broad assessment of the quality of MAs in depression using widely accepted standards is of greater necessity than similar assessments of MAs in other fields. However, the reporting or methodological qualities of current MAs in depression using PRISMA or AMSTAR remain uninvestigated. This gap of knowledge may lessen the confidence of clinicians and policy makers in the mental health area in the process of decision-making based on evidence from existing MAs. The present study was performed to evaluate the research quality of MAs in depression.

Due to the large amount of MAs publications in psychiatry, we specifically focus on studies pertinent to depression that were published in the years of 2014 and 2015, which were assumed to represent the current quality in this field. We expect to assess the reporting and methodological qualities of included MAs by using PRISMA and AMSTAR checklists and identify potential predictive factors associated with the quality of studies.

## Methods

### Search strategy

Comprehensive searches were performed in PubMed (MEDLINE), EMBASE, PsycINFO and Cochrane Library for MAs published in peer-reviewed journals from January 2014 to December 2015 with the language limited to English. A meta-analysis is defined as “a statistical analysis combining results from independent studies, which produces a single estimate of effect, using appropriate pooling methods (e.g. the random- or fixed-effect models)” [14]. Duplicated publications, abstracts only and conference proceedings were excluded. In PubMed, EMBASE and PsycINFO, a search strategy with the combination of free words "meta?analy*" and "depress*", as well as MeSH word "Depression" and publication type of "meta-analysis". Studies investigating epidemiology and intervention for clinical depression, major depression, unipolar depression, recurrent depression and postpartum depression, along with major depressive disorder (MDD), were included. Bipolar depression and depressive symptoms were excluded. The search was finished in April 2016.

### Eligibility criteria and study selection

In the present study, inclusion criteria were 1) studies pooling results from primary studies with the meta-analytic methodology (e.g. MAs alone or systematic review with MAs); 2) studies assessing the epidemiology, basic research and clinical therapies of depression and 3) articles written in English. Exclusion criteria were 1) studies that are not MAs in nature, such as narrative reviews, expert opinions or comments and 2) MAs evaluating diseases other than depression (e.g. schizophrenia or anxiety disorder). After duplications being removed, four investigators (YBZ, LF, XCM and HZ) independently reviewed the titles and abstracts of the identified studies. Full-text articles were then retrieved for potentially eligible studies. Discrepancies were resolved through discussion and consultation with another author (YS).

### Data extraction

Two reviewers (YBZ and LF) independently extracted the general characteristics of the included studies, which include author and publishing journal information, as well as characteristics of the MAs.

Author information included 1) the corresponding author’s original region and country, 2) the corresponding author’s affiliations, 3) number of authors and their employers, and 4) presence of co-authors from epidemiology or statistics departments. In addition, to evaluate the first author’s expertise in conducting MAs, we specially performed a PubMed search to investigate the number of MAs publications the first author had previously co-authored (i.e. the first author’s previous experience).

Published journal information included 1) journal name and impact factor according to the 2014 Journal Citation Report (JCR) from Thomson ISI (Institute for Scientific Information), 2) whether open access or not according to the Open Access Journal List from Thomson Reuters [15], 3) journal’s ranking in its subject category, obtained from the latest data of SCImago Journal & Country Rank indicator (ranking journals from the first to fourth quartile (Q1 to Q4) in its respective subject category) [16] and 4) journal’s requirement of adherence to PRISMA for publication (PRISMA endorsement), obtained from the author’s instructions for each journal.

The characteristics of MAs were the following: 1) topic of the study, such as basic research, drug therapy and psychotherapy, *etc*., 2) numbers of included studies and participants, 3) numbers of included randomized controlled trials (RCTs), 4) funding sources, 5) registration status, which referred to whether the study was prospectively registered on International Prospective Register of Systematic Reviews (PROSPERO) [17], 6) pooling method(s) used to combine data and 7) positive or negative tendency of the conclusions based on their primary endpoints (preventions or interventions with significant effects were cited as positive, and those with no effect or controversial results were cited as negative).

### Assessment of reporting and methodological quality

Reporting quality of eligible MAs was assessed using PRISMA statement. Each item was chosen based on empirical evidence for being important in a completely written report of MAs. The PRISMA statement is a list of 27 items used to assess whether the publication reported all relevant information. Each PRISMA item is rated with a “yes” or “no” response [8].

The methodological quality was evaluated using AMSTAR. AMSTAR is an 11-item questionnaire assessing whether the methods were appropriately used and reduction of bias existed in MAs. In particular, content and construct validity as well as inter-rater reliability were assessed independently in AMSTAR. Each individual item is rated with a “yes” (scored 1 point), “no” (scored 0 points) or “cannot answer” (scored 0 points) [11]. A “yes” response means that the item is fulfilled; a “no” response means that the item is not fulfilled and a “cannot answer” response means that it is inconclusive whether the item is fulfilled. Each MAs was separately and independently assessed by two authors (YBZ and HZ), who were blinded to the author and journal information. Disagreements were resolved through discussion.

### Data analysis

Data were put into a spreadsheet program (Microsoft Excel 2013, Microsoft) and analyzed by statistical software SPSS (version 21.0, IBM, Chicago). Level of concordance of reviewers was measured by *kappa* value. A value of 0.65 or greater was chosen for sufficient agreement. Dichotomous factors affecting the study qualities were analyzed using independent ***t*** test while multifactorial variables were compared using one-way analysis of variance (ANOVA), in which differences between two factors were compared with the Dunnett ***t*** test. Pearson correlation was used to detect the potential consistency between reporting quality (determined by PRISMA score) and methodologic quality (determined by AMSTAR score). Univariate linear regression analysis was used to identify factors with potential influence on study qualities; factors with significance less than 0.25 were entered into subsequent stepwise multivariate linear regression. *P* values less than 0.05 were considered significant on statistical analyses.

## Results

### Search results

**Fig 1:**
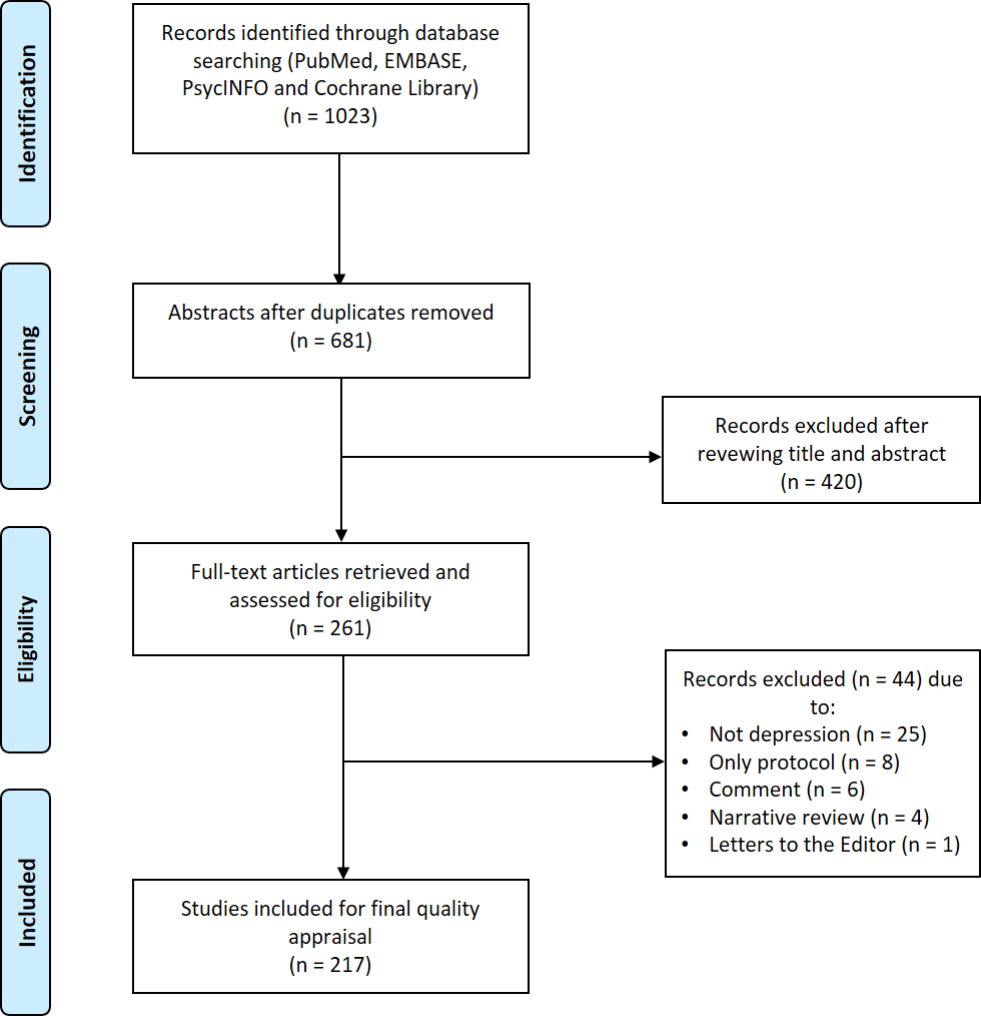
Flow diagram of meta-analysis inclusion.

The search protocol identified 1023 studies with potential relevance. After screening of titles and abstracts, 261 studies were eligible for full-text review. Forty-four of them were excluded according to exclusion criteria. Thus, 217 MAs from 74 peer-reviewed journals were entered for quality assessment (Fig 1**)**. Information of the 74 peer-reviewed journals is listed in S1 Table.

### General characteristics

Characteristics of all included MAs are shown in Table 1. Nearly half of the assessed MAs came from Europe (43.3%, 94/217), and the largest part (17.5% (38/217) of the included MAs was conducted by author from the United Kingdom. The mean impact factor of all publishing journals was 5.0 and the median author number was five. Most journals (90.3%, 196/217) were not open-access, 85.7% (186/217) were ranked as Q1 defined by SCImago Journal & Country Rank, but only 14.8% (32/217) had PRISMA endorsement. Less than half (42.9%, 93/217) of the studies enrolled only primary studies with RCT design in their MAs. In addition, only 12% (26/217) of the included studies had registered before publication on the PROSPERO database. The median number of participating authors was five, among which epidemiologists or statisticians were involved in 11.1% (24/217) of studies. Most of the correspondent authors (91.2%, 198/217) were from university-affiliated units and less than half (43.3%, 94/217) of them had previous experience publishing at least one MA.

**Table 1:** Characteristics of included meta-analyses.

Characteristics	(%)	N
**Year of publication**		
2014	49.8	108
2015	50.2	109
**Topic**		
Basic research	18.9	41
Drug therapy	25.8	56
Psychotherapy	12.9	28
Procedure	14.3	31
Diagnostic/Epidemiologic	5.5	12
Other	22.6	49
**Continent**		
North America	21.2	46
Europe	43.3	94
Asia and Oceania	30.4	66
Africa/South America	4.6	10
**Country or region of origin**		
UK	17.5	38
China	14.7	32
USA	14.3	31
Canada	9.2	20
Netherlands	7.4	16
Australia	6.5	14
Germany	6.0	13
Other	24.4	53
**Journal impact factor**		
Lower (<5.0)	54.8	119
Higher (≥5.0)	35.5	77
N/A	9.7	21
Journal rank*		
Q1	85.7	186
Q2 or Q3	13.4	29
Unranked	0.9	2
**Open access journal**		
No	90.3	196
Yes	9.7	21
**Affiliation of the correspondent author**		
Non-university affiliated	8.8	19
University affiliated	91.2	198
**PRISMA endorsement**		
No	85.2	185
Yes	14.8	32
First author’s experience**		
No	56.7	123
Yes	43.3	94
**Statistician as coauthor**		
No	88.9	193
Yes	11.1	24
**Registration**		
No	88.0	191
Yes	12.0	26
**Participating center**		
Single	15.2	33
Multiple	84.8	184
**Funding support**		
No	49.3	107
Yes	50.7	110
**Number of included studies**		
<20	65.0	141
≥20	35.0	76
**Number of authors**		
<5	33.2	72
≥5	66.8	145
**Design of included studies**		
RCT only	42.9	93
RCT and observational studies	57.1	124
**Use of pooling methods**		
Random or fixed effect	77.9	169
Both	18.0	39
N/A	4.1	9
**Conclusion on primary outcome**		
Negative	18.9	41
Positive	79.7	173
N/A	1.4	3
**Cochrane or not**		
No	92.2	200
Yes	7.8	17

* Rankings of the journals in each subspecialty were obtained from the latest data (2013) from SCImago Journal Rank (SJR) indicator. Q1-Q3 indicates the first to third quartiles.

** First author’s experience of having previously co-authored more than one published meta-analysis.

**Abbreviations:** N/A, Not available; AMSTAR, Assessment of Multiple Systematic Reviews; PRISMA, Preferred Reporting Items for Systematic Reviews and Meta-Analyses; RCT, Randomized controlled trial; SD, Standard deviation.

### Reporting quality

**Fig 2:**
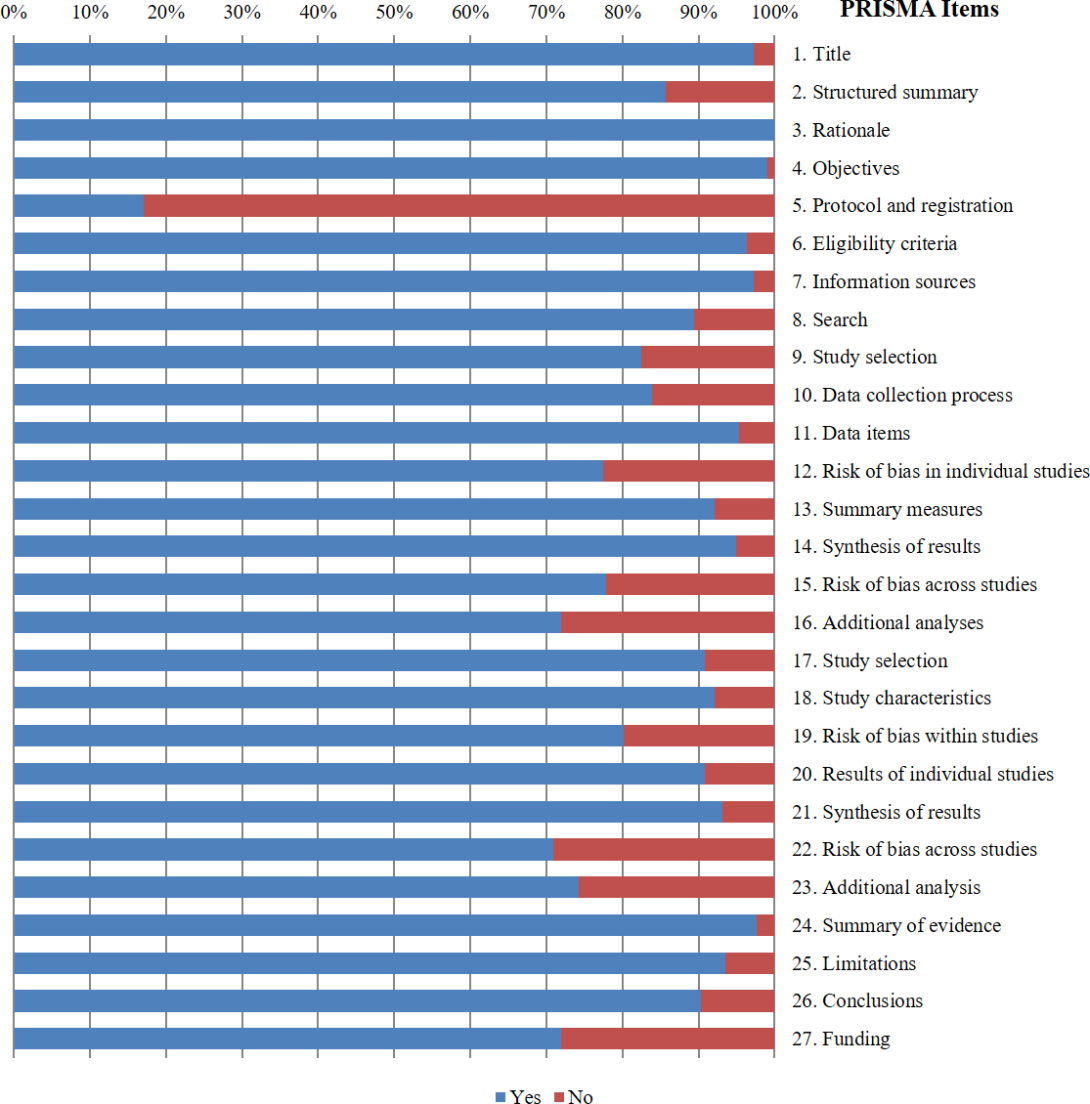
Individual items of and adherence to the PRISMA checklist. A higher percentage indicates a better adherence. “Yes” means that the item is fulfilled; “No” means that the item is not fulfilled or unavailable.

Assessment of reporting quality using PRISMA statement began after agreement among reviewers was substantial [*kappa* = 0.75, 95% confidence interval (CI), 0.69~0.82]. The mean PRISMA score of all included 217 MAs was 23.0±3.5 out of 27. The mean adherence rate of all items to the checklist was 84.6%, which indicated a moderate reporting quality (Fig 2). The item assessing registration and protocol (Item 5) had the poorest adherence rate (14.2%, 37/217), followed by the item assessing risk of bias (Item 12) within studies (70.6%, 156/217). The item assessing rationale of the study (Item 3) had the best adherence rate to the checklist (100%). We further divided MAs into certain subgroups according to their characteristics and compared their reporting and methodology qualities using PRISMA and AMSTAR scores. As is shown in Table 2, the MAs that are pre-registered, included only RCTs, had negative results at primary outcome and MAs being searched from Cochrane library had better reporting and methodological quality (Table 2).

**Table 2:** Characteristics of included MAs and comparison of their qualities.

Features	PRISMA score (Mean±SD)	*P* value	AMSTAR score (Mean±SD)	*P* value
**Year of publication**				
2014	23.5±2.5	0.053	8.7±1.3	0.082
2015	22.5±4.3		7.8±2.0	
**Journal impact factor**				
Lower (<5.0)	23.2±3.2	0.993	8.3±1.7	0.897
Higher (≥5.0)	23.2±2.8		8.3±1.5	
Journal rank*				
Q1	23.0±3.6	0.987	8.3±1.7	0.975
Q2 or Q3	23.0±3.2		8.3±1.4	
**Open access journal**				
No	22.9±3.6	0.659	8.2±1.7	0.733
Yes	23.2±2.9		8.4±1.7	
**Affiliation of the correspondent author**				
Non-university affiliated	22.9±2.2	0.904	8.3±1.2	0.831
University affiliated	23.0±3.6		8.3±1.8	
**PRISMA endorsement**				
No	22.8±3.7	0.155	8.2±1.8	0.285
Yes	23.8±2.6		8.6±1.5	
First author’s experience**				
No	22.7±4.1	0.154	8.2±1.9	0.444
Yes	23.4±2.6		8.4±1.6	
**Statistician as coauthor**				
No	22.9±3.4	0.715	8.2±1.7	0.551
Yes	23.2±4.5		8.5±2.2	
**Pre-registration**				
No	22.7±3.6	**0.009**	8.1±1.8	**0.001**
Yes	24.7±2.2		9.3±1.2	
**Participating center**				
Single	22.5±3.3	0.405	8.5±1.4	0.358
Multiple	23.0±3.0		8.2±1.8	
**Funding support**				
No	22.9±3.7	0.862	8.2±1.7	0.503
Yes	23.0±3.4		8.3±1.8	
**Number of included studies**				
<20	23.0±3.3	0.995	8.2±1.7	0.846
≥20	23.0±3.9		8.3±1.7	
**Number of authors**				
<5	22.1±3.7	**0.009**	8.0±1.5	0.168
≥5	23.4±3.4		8.4±1.8	
**Design of included studies**				
RCT only	23.7±2.3	**0.004**	8.7±1.5	**0.003**
RCT and observational studies	22.4±4.2		8.0±1.9	
**Use of pooling methods**				
Random or fixed effect	22.9±3.6	0.452	8.2±1.7	0.173
Both	23.4±3.2		8.6±1.7	
**Conclusion on primary endpoint**				
Negative	23.5±2.4	0.268	9.1±1.1	**<0.001**
Positive	22.9±3.7		8.1±1.8	
Cochrane or not***				
No	22.8±3.6	**0.019**	8.1±1.7	**<0.001**
Yes	24.9±1.4		9.8±0.4	

* Rankings of the journals in each subspecialty were obtained from the latest data (2013) from SCImago Journal Rank (SJR) indicator. Q1-Q3 indicated the first to third quartiles.

** First author’s experience means whether the first author had previous experience of publishing at least one meta-analysis.

*** Cochrane or not means whether MAs included in present study were from Cochrane database.

*P* values refer to *t*-test between subgroups of each characteristic.

**Abbreviations:** AMSTAR, Assessment of Multiple Systematic Reviews; PRISMA, Preferred Reporting Items for Systematic Reviews and Meta-Analyses; RCT, Randomized controlled trial; SD, Standard deviation.

### Methodological quality

**Fig 3:**
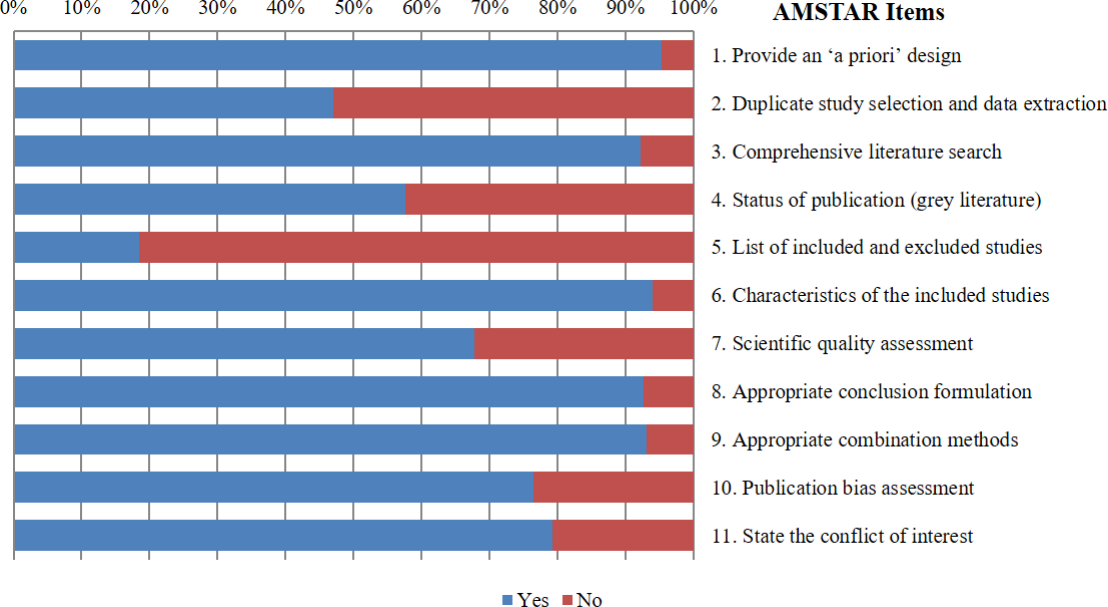
Individual items of and adherence to the AMSTAR checklist. A higher percentage indicates a better adherence. “Yes” means that the item is fulfilled; “No” means that the item is not fulfilled or unavailable.

**Fig 4:**
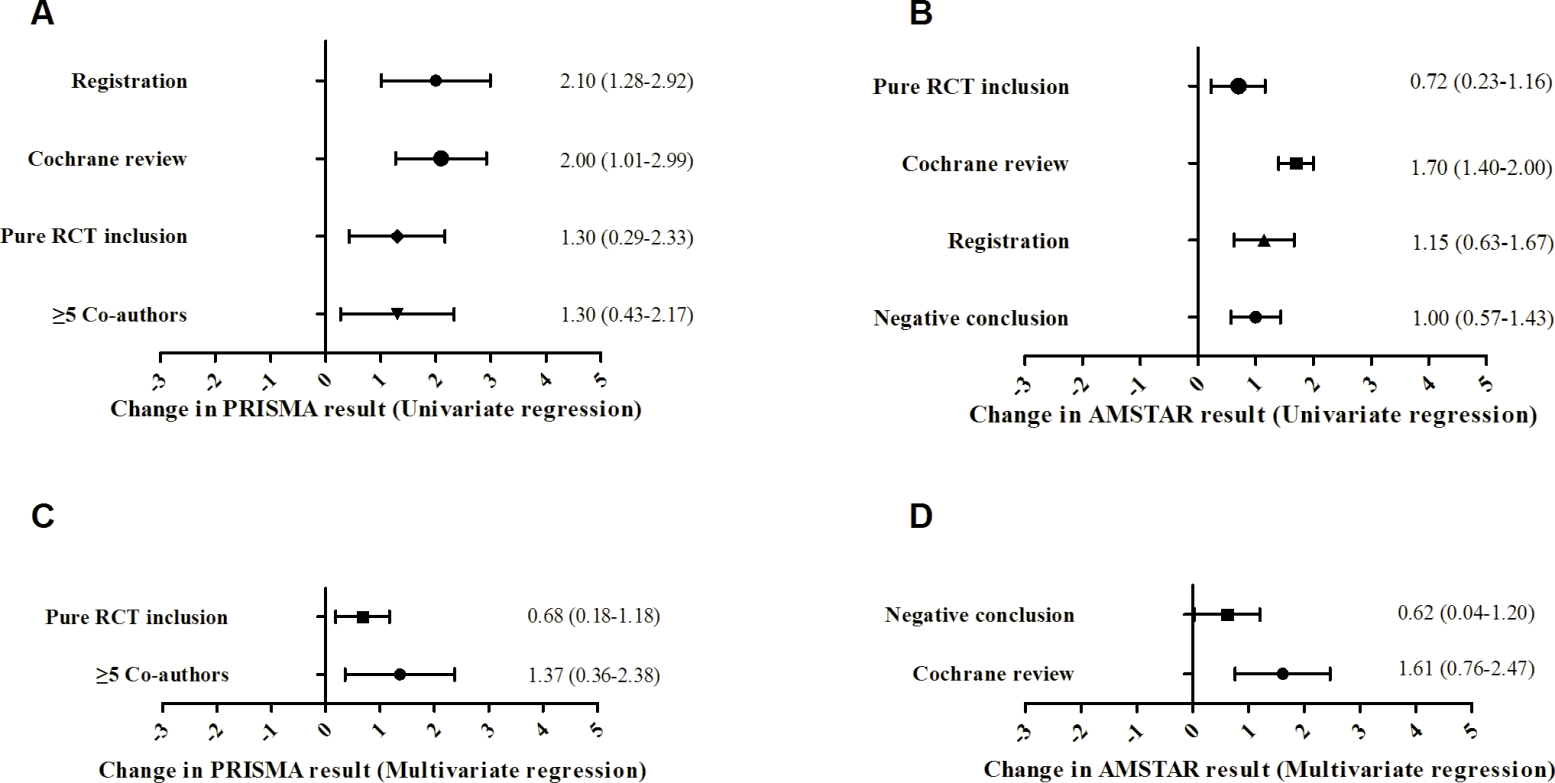
Scatter graph of AMSTAR and PRISMA results with a linear model. A deeper color indicates more dense points. R refers to the correlation coefficient.

The methodological quality was assessed using AMSTAR, and the agreement among reviewers was substantial (*kappa* = 0.71, 95% CI, 0.63~0.84). The mean AMSTAR score of all included MAs was 8.3±1.7 out of 11. The mean adherence rate of all items to AMSTAR was 73.1%, which indicated a moderate overall methodological quality. The item requiring a full list of included and excluded studies (Item 5) had the poorest adherence rate (16.1%, 40/217), followed by the item concerning duplicate study selection and data extraction (Item 2, 45.0%, 102/217). The item concerning providing an ‘a priori’ design (Item 1) was found to have the highest adherence rate (94.8%, 207/217) (Fig 3). In addition, PRISMA scores had a linear correlation with those of AMSTAR (*R*^2^ = 0.56, *P* < 0.001) (Fig 4).

### Association of variables and study quality

**Fig 5:**
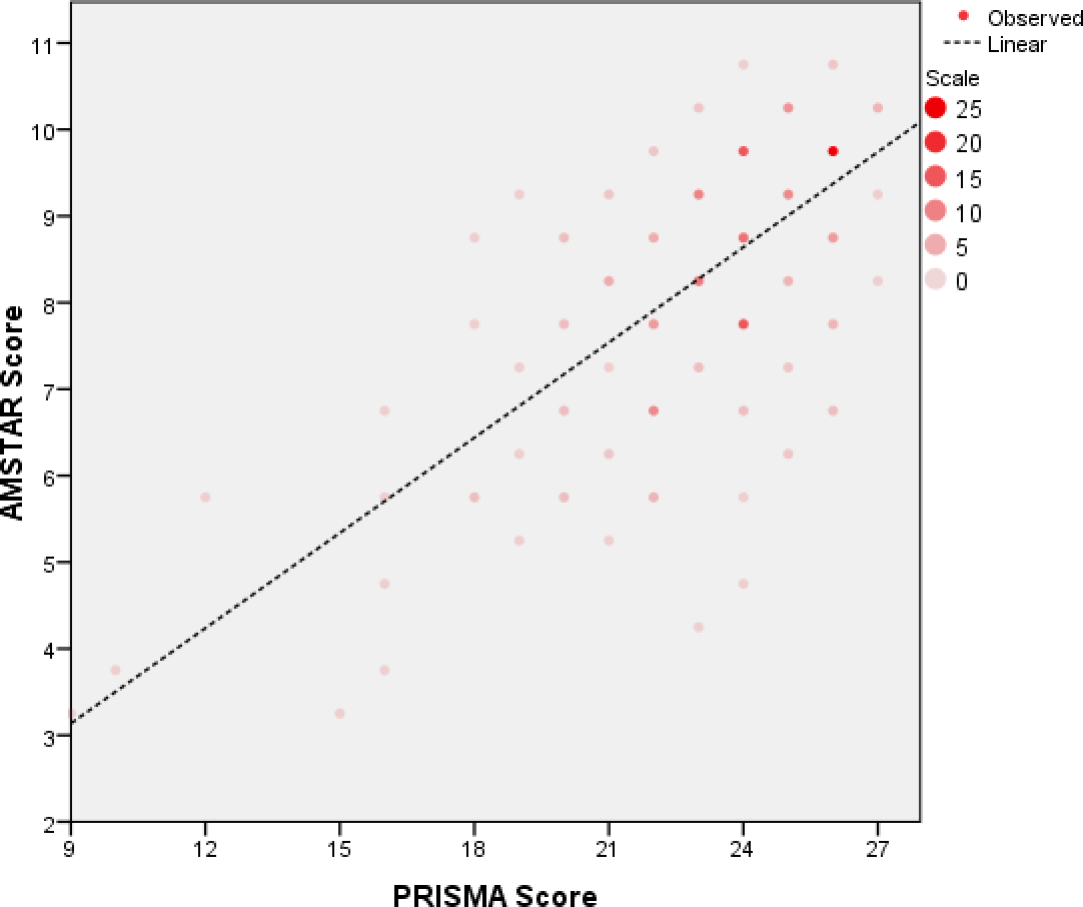
Forest plots of the changes in PRISMA and AMSTAR scores for factors included in univariate and multivariate regression models, along with 95% confidence intervals. A, univariate linear regression of different factors for PRISMA result, all variables were binary; B, univariate linear regression of different factors for AMSTAR result, all variables were binary; C, results of multivariate regression. Factors with *P* < 0.25 in univariate analysis were entered into multivariate analysis. After adjustment, only one variable was significantly associated with the PRISMA result; D, results of multivariate regression. Factors with *P* < 0.25 in univariate analysis were entered into multivariate analysis. After adjustment, only one variable was significantly associated with the AMSTAR result.

Potential factors affecting the quality of MAs are listed in **Table 2.** Factors associated with higher PRIMSA score included pre-registration (*P* = 0.009), only RCT included (*P* = 0.004), more than five authors (*P* = 0.009) and authors from Cochrane group (*P* = 0.019). Factors associated with higher AMSTAR score included pre-registration (*P* = 0.001), only RCT included (*P* = 0.003), finding negative results (*P* < 0.001) and authors from Cochrane group (*P* < 0.001). These factors were selected for multivariate regression analysis (Fig 5). After adjustment for other variables, only RCT included and more than five authors were found to independently increase the total PRISMA score by 0.68 points (95% CI: 0.18~1.18, *P* = 0.010) and 1.37 points (95% CI: 0.36~2.38, *P* = 0.008), respectively. The MAs found negative results and had authors from Cochrane group had higher AMSTAR score by 0.62 points (95% CI: 0.04~1.20, *P* = 0.037) and 1.61 points (95% CI: 0.76~2.47, *P* < 0.001), respectively (Fig 5). Other factors were not correlated with either reporting or methodological quality, including impact factor, rank, PRISMA endorsement, affiliated to universities, statistician included as co-authors, single or multiple centers, funding support, numbers of included studies or patients, pooling methods and publishing experience of the first author.

## Discussion

In the present study, we reviewed 217 MAs pertinent to depression published in years 2014 and 2015, and found that compliances to the PRISMA statement and AMSTAR checklist remained to be improved. The overall qualities were 84.6% and 73.1% in reporting and methodology, respectively. We found that MAs with pre-registration, only RCT included and authors from Cochrane groups had better reporting and methodological qualities. The number of authors was associated with better quality of report and negative result of MAs was associated with better quality of methodology. To our knowledge, this is the first study to examine the research quality of MAs of depression using PRISMA statement and AMSTAR tools.

Previous studies evaluated the methodological quality of MAs in depression 50.2% [18–21] and anxiety 57.2% [22] using QUOROM checklist. In addition, the reporting quality of MAs in other fields e.g., radiology, and neurosurgery ranged between 31.1% and 85.2% using PRISMA; while the methodological quality of MAs in these references ranged between 47.3% and 72.7% using AMSTAR [6,12,19,21,23,24]. However, the quality evaluation of MAs in the field of depression using above tools remain uninvestigated. In the present study, the reporting quality of MAs pertinent to depression is 85.2% and the methodological quality is 75.5%, which are consistent or higher than qualities reported by previous studies above. The higher quality of MAs found in present study may be attributed to wider adoption of the publication guidelines and better awareness of the quality criteria. However, there are still some items in the checklists that are poorly met, calling for greater awareness of future investigators.

A number of PRISMA items were well reported in the included MAs of depression. Almost all included studies provided a sufficient rationale and objective for their analysis (100% adherence to item 3 and 98.6% to item 4), which allow readers to quickly understand the scientific background. Another well-adhered item, a summary of the authors’ findings in the discussion section (item 24, with 97.2% adherence), provides readers with a quick glance at the main outcomes of the study before extended discussions. These items with good adherence indicate that all included studies have properly adopted the conventional criteria in scientific writing, such as the ICMJE publication recommendations [25]. In addition, the majority of studies provided details on information sources, data items evaluated, and pooling methods of the results, represented by high compliances of three items (items 7, 11 and 14). It indicates that most of the studies reported the core elements of MAs sufficiently and used the pooling methods appropriately [26].

The poorest reported item was the one requiring protocol or registration information (Item 5) of MAs. In our study, only 14.2% of the included studies adhered to this item. It is highly recommended for MAs to include the study with protocol addressing specific questions, e.g., trial inclusion and exclusion criteria, the methodological approach to be taken, and analyses that are planned [27]. Meanwhile, registration on open platforms (e.g., PROSPERO) is a practical way to prevent duplications and selective outcome-reporting biases of systematic review and MAs [17]. Therefore, pre-registration on open platform with a prospective protocol should be considered for future authors prior to conduction of MAs.

The methodological quality of MAs in depression found in the present study was assessed using the AMSTAR checklist. We found that most of the included studies provided “a priori’ design” (Item 1, adherence = 94.8%) and detailed characteristics of the included studies (Item 6, adherence = 93.4%). It suggests that these studies have provided explanation of background and settings, inclusion and exclusion criteria, demographic and socioeconomic status of the participants, interventions and outcomes sufficiently. This enables readers to better understand the study topic and interpret the results and contexts of the MAs properly. Interestingly, a large portion of included studies (91.5%) had conducted a comprehensive literature search (item 3), which is defined as two or more electronic sources being searched with a description of publication years, databases used and search strategy. Substantial improvement in this methodology was identified in the present assessment compared with previous studies [6,12,19]. Since a comprehensive search for all available evidence is essential for MAs, it is quite important for authors to be rigorous in the process of data search. However, there is poor adherence to several items of the AMSTAR checklist. Less than half (45.0%) of studies assessed duplicate selection and data extraction process (Item 2), which requires at least two independent data extractors and a consensus procedure for disagreements. A lack of mutual agreement and discussion may introduce selection bias and other confounders, which may further impair the power of the pooled results of the MAs. Additionally, only a minority of studies (16.1%) provided a list of included and excluded studies (item 5), which requires a table/list/figure of included and excluded studies, either in text or in supplemental sources [28]. Failing to report the detailed study inclusion and exclusion process may increase the risk of selective reporting, which causes bias in MAs. Therefore, future investigators should note that a clear and complete study selection process is essential in checking for any missed or erroneously included studies.

Characteristics associated with overall reporting and methodological qualities were explored. After adjustment for confounders, MAs conducted by multiple (over five) authors and those with only RCT included had higher PRISMA scores (1.4 and 0.7 points respectively, out of a maximum of 27) than MAs without such characteristics. This subtle superiority may suggest that the cooperation of authors can be beneficial, which is common in the complex work including the preparation of MAs. It remains controversial whether observation studies should be included other than RCTs, and whether the poor methodology of included studies would impair the quality of MAs [29, 30]. In present study, we found that only including RCTs is associated with better quality of MAs. Since it is the first study to evaluate the quality of MAs in the field of depression, this result is pending on further validation in the future.

MAs conducted by Cochrane group had superior methodological quality (1.6 points higher out of the 11-point AMSTAR checklist) than MAs conducted by non-Cochrane group authors, which is consistent with previous studies in other fields [6,31,32]. It indicates that strict training of methodologies and collaborative guidance among experts is beneficial for producing high quality MAs [20]. Meanwhile, we found that the previous experience of publication of the first author had no effect on quality of MAs. As we know, the Cochrane group requires specific training with rigorous standards for methodology. It is more likely the professional training of Cochrane group, instead of previous experience of publishing papers that attributes to high quality of MAs.

Interestingly, we found negative findings of MAs can be an independent factor for better methodology (0.6 points higher in AMSTAR score). It may be attributed to the common favor of positive results by journals. As we know, it is rather difficult to get published if a study reported negative results [33]. Therefore, MAs with negative results tend to utilize relatively more rigorous criteria to include more negative trials for final analysis.

Previous studies have reported that external funding source may introduce bias and influence the conclusion of clinical studies towards an interest-related way [34]. Given that 50.7% (n = 110) of included studies being supported by funding, we further examined whether external funding source influence the quality of MAs. No association was found between existence of funding sources and reporting as well methodological qualities.

There are some limitations in the present study. First, we only included MAs written in English, and this language restriction may subject the study to potential publication bias. Second, assessment of reporting quality depends on the descriptions by authors, which may not be consistent with what has actually been done. Third, although the kappa values of reviewers indicated substantial inter-rater agreement, minor discrepancies between investigators remain existed and may introduce potential bias into the present study. Despite these limitations, this study evaluates the need for improving quality of MAs in the field of depression. It was the first study, to our knowledge, to evaluate reporting and methodological qualities independently using two latest tools.

In conclusion, quality of MAs in the field of depression is improving with more and more rigorous requirements of submission by journals. But the overall quality remains to be improved. Design of included studies, characteristics of authors and pre-registration of MAs are important factors which may influence quality of MAs. Particularly, MAs with only RCTs included, conducted by more authors (over five) and authors from Cochrane group may achieve better reporting and methodological qualities. These findings are indicative for authors and readers of MAs in the future.

## Supporting Information

S1 Table Publishing journals of the included meta-analyses. (DOCX)

S2 Table A self-rated PRISMA checklist for the present study. (DOCX)

S3 Table Complete search strategy used in PubMed database. (DOCX)
