# Supplementary material for: Is the Best Evidence Good Enough: Quality Assessment and Factor Analysis of Meta-Analyses on Depression
Source: PLoS One. 2016 Jun 23;11(6):e0157808. doi: 10.1371/journal.pone.0157808 (PMC4919061; doi:10.1371/journal.pone.0157808)
Supplement: S1 Table — (DOCX) [file pone.0157808.s001.docx]

**S1 Table.** Publishing journals of the included meta-analyses.

| No. | **Name of Published Journals** | **Impact Factor (2014)** | **Subject category** | **Ranking** | **Number of included meta-analyses** |
| --- | --- | --- | --- | --- | --- |
| 1 | *Acta neuropsychiatrica* | 0.802 | Psychiatry and Mental Health | Q3 | 1 |
| 2 | *Acta psychiatrica Scandinavica* | 5.605 | Psychiatry and Mental Health | Q1 | 2 |
| 3 | *American journal of medical genetics* | 3.416 | Genetics (clinical) | Q2 | 1 |
| 4 | *American journal of kidney diseases* | 5.9 | Nephrology | Q1 | 1 |
| 5 | *American journal of psychiatry* | 12.295 | Psychiatry and Mental Health | Q1 | 3 |
| 6 | *Annals of clinical psychiatry* | 2.364 | Psychiatry and Mental Health | Q2 | 1 |
| 7 | *Asia-Pacific psychiatry* | 0.63 | Psychiatry and Mental Health | Q3 | 2 |
| 8 | *Biological psychiatry* | 10.255 | Biological Psychiatry | Q1 | 2 |
| 9 | *BMC medicine* | 7.249 | Medicine | Q1 | 1 |
| 10 | *BMC psychiatry* | 2.21 | Psychiatry and Mental Health | Q1 | 8 |
| 11 | *BMJ* | 17.445 | Medicine | Q1 | 1 |
| 12 | *BMJ open* | 2.271 | Medicine | Q1 | 3 |
| 13 | *Brain, behavior, and immunity* | 5.889 | Behavioral Neuroscience | Q1 | 1 |
| 14 | *Clinical psychology review* | 7.179 | Psychiatry and Mental Health | Q1 | 4 |
| 15 | *CNS drugs* | 5.113 | Psychiatry and Mental Health | Q1 | 1 |
| 16 | *Cochrane database of systematic reviews* | 6.032 | Medicine | Q1 | 17 |
| 17 | *Depression and anxiety* | 4.407 | Psychiatry and Mental Health | Q1 | 1 |
| 18 | *European child & adolescent psychiatry* | 3.336 | Psychiatry and Mental Health | Q1 | 4 |
| 19 | *European neuropsychopharmacology* | 4.369 | Psychiatry and Mental Health | Q1 | 4 |
| 20 | *European Psychiatry* | 3.439 | Psychiatry and Mental Health | Q1 | 1 |
| 21 | *Expert opinion on drug safety* | 2.911 | Pharmacology | Q2 | 1 |
| 22 | *General hospital psychiatry* | 2.606 | Psychiatry and Mental Health | Q2 | 4 |
| 23 | *Human psychopharmacology* | 2.192 | Psychiatry and Mental Health | Q2 | 2 |
| 24 | *Indian journal of psychiatry* | - | Psychiatry and Mental Health | Q3 | 1 |
| 25 | *International clinical psychopharmacology* | 2.456 | Psychiatry and Mental Health | Q1 | 1 |
| 26 | *International journal of geriatric psychiatry* | 2.866 | Psychiatry and Mental Health | Q1 | 2 |
| 27 | *International psychogeriatrics* | 1.934 | Psychiatry and Mental Health | Q2 | 2 |
| 28 | *JAMA internal medicine* | 13.116 | Medicine | Q1 | 1 |
| 29 | *JAMA* | 35.289 | Medicine | Q1 | 1 |
| 30 | *JAMA psychiatry* | 12.008 | Psychiatry and Mental Health | Q1 | 5 |
| 31 | *Journal of abnormal child psychology* | 3.09 | Psychiatry and Mental Health | Q1 | 1 |
| 32 | *Journal of acquired immune deficiency syndromes* | 4.556 | Pharmacology | Q1 | 1 |
| 33 | *Journal of affective disorders* | 3.383 | Psychiatry and Mental Health | Q1 | 32 |
| 34 | *Journal of cerebral blood flow and metabolism* | 5.407 | Nephrology | Q1 | 1 |
| 35 | *Journal of ethnopharmacology* | 2.998 | Pharmacology | Q1 | 1 |
| 36 | *Journal of medical Internet research* | 3.428 | health informatics | Q1 | 1 |
| 37 | *Journal of neurology, neurosurgery, and psychiatry* | 6.807 | Psychiatry and Mental Health | Q1 | 1 |
| 38 | *Journal of psychiatric research* | 3.957 | Psychiatry and Mental Health | Q1 | 7 |
| 39 | *Journal of psychiatry & neuroscience* | 5.861 | Psychiatry and Mental Health | Q1 | 1 |
| 40 | *Journal of psychopharmacology* | 3.593 | Psychiatry and Mental Health | Q1 | 1 |
| 41 | *Journal of psychosomatic research* | 2.736 | Psychiatry and Mental Health | Q2 | 2 |
| 42 | *Journal of the American Academy of Child and Adolescent Psychiatry* | 7.26 | Psychiatry and Mental Health | Q1 | 1 |
| 43 | *Molecular psychiatry* | 14.496 | Psychiatry and Mental Health | Q1 | 2 |
| 44 | *Neuropsychiatric disease and treatment* | 1.741 | Psychiatry and Mental Health | Q2 | 2 |
| 45 | *Neuroscience and biobehavioral reviews* | 8.802 | Neuropsychology and Physiological Psychology | Q1 | 5 |
| 46 | *Obstetrics and gynecology* | 5.175 | Obstetrics and Gynecology | Q1 | 1 |
| 47 | *Pharmacogenomics* | 3.218 | Pharmacology | Q2 | 1 |
| 48 | *Pharmacopsychiatry* | 1.851 | Psychiatry and Mental Health | Q1 | 1 |
| 49 | *PLoS medicine* | 14.429 | Medicine | Q1 | 1 |
| 50 | *PLoS One* | 3.234 | Medicine | Q1 | 4 |
| 51 | *Progress in neuro-psychopharmacology & biological psychiatry* | 3.689 | Biological Psychiatry | Q2 | 2 |
| 52 | *Psychiatric genetics* | 1.941 | Psychiatry and Mental Health | Q2 | 1 |
| 53 | *Psychiatry and clinical neurosciences* | 1.634 | Psychiatry and Mental Health | Q2 | 2 |
| 54 | *Psychiatry investigation* | 1.28 | Psychiatry and Mental Health | Q2 | 1 |
| 55 | *Psychiatry research* | 2.467 | Psychiatry and Mental Health | Q1 | 5 |
| 56 | *Psychological bulletin* | 14.756 |  |  |  |
| 57 | *Psychological medicine* | 5.938 | Psychiatry and Mental Health | Q1 | 13 |
| 58 | *Psychoneuroendocrinology* | 4.944 | Psychiatry and Mental Health | Q1 | 5 |
| 59 | *Psychopharmacology* | 3.875 | Pharmacology | Q1 | 3 |
| 60 | *Psychosomatics* | 1.858 | Psychiatry and Mental Health | Q1 | 1 |
| 61 | *Psychotherapy and psychosomatics* | 9.196 | Psychiatry and Mental Health | Q1 | 1 |
| 62 | *Rheumatology* | 4.475 | Pharmacology | Q1 | 1 |
| 63 | *Schizophrenia bulletin* | 8.45 | Psychiatry and Mental Health | Q1 | 1 |
| 64 | *Scientific reports* | 5.578 | Multidisciplinary | Q1 | 1 |
| 65 | *Social psychiatry and psychiatric epidemiology* | 2.537 | Psychiatry and Mental Health | Q1 | 2 |
| 66 | *Sports medicine* | 5.038 | Medicine | Q1 | 1 |
| 67 | *The American journal of geriatric psychiatry* | 4.235 | Psychiatry and Mental Health | Q1 | 3 |
| 68 | *The Australian and New Zealand journal of psychiatry* | 3.407 | Psychiatry and Mental Health | Q1 | 3 |
| 69 | *The British journal of psychiatry* | 7.991 | Psychiatry and Mental Health | Q1 | 4 |
| 70 | *The international journal of neuropsychopharmacology* | 4.009 | Psychiatry and Mental Health | Q1 | 5 |
| 71 | *The Journal of clinical psychiatry* | 5.498 | Psychiatry and Mental Health | Q1 | 9 |
| 72 | *The world journal of biological psychiatry* | 4.183 | Biological Psychiatry | Q2 | 3 |
| 73 | *Therapeutic advances in psychopharmacology* | 1.53 | Pharmacology | Q2 | 1 |
| 74 | *World psychiatry* | 14.225 | Psychiatry and Mental Health | Q1 | 3 |
