# Supplementary material for: Is the Best Evidence Good Enough: Quality Assessment and Factor Analysis of Meta-Analyses on Depression
Source: PLoS One. 2016 Jun 23;11(6):e0157808. doi: 10.1371/journal.pone.0157808 (PMC4919061; doi:10.1371/journal.pone.0157808)
Supplement: S3 Table — (DOCX) [file pone.0157808.s003.docx]

**S3 Table**. Complete search strategy used in PubMed database

| **Search** | **Query** |
| --- | --- |
| #1 | ("depressive disorder"[MeSH Terms] OR ("depressive"[All Fields] AND "disorder"[All Fields]) OR "depressive disorder"[All Fields] OR "depression"[All Fields] OR "depression"[MeSH Terms]) AND (Meta-Analysis[ptyp]) |
| #2 | (depress[All Fields] OR depressa[All Fields] OR depressability[All Fields] OR depressable[All Fields] OR depressan[All Fields] OR depressana[All Fields] OR depressant[All Fields] OR depressant'[All Fields] OR depressantami[All Fields] OR depressantov[All Fields] OR depressants[All Fields] OR depressants'[All Fields] OR depressao[All Fields] OR depressaria[All Fields] OR depressariidae[All Fields] OR depresse[All Fields] OR depressed[All Fields] OR depressed'[All Fields] OR depressed''[All Fields] OR depressedosteoblast[All Fields] OR depresseed[All Fields] OR depressei[All Fields] OR depresseion[All Fields] OR depressent[All Fields] OR depresser[All Fields] OR depresses[All Fields] OR depressesed[All Fields] OR depressess[All Fields] OR depresseur[All Fields] OR depresseurs[All Fields] OR depresseux[All Fields] OR depressi[All Fields] OR depressi'[All Fields] OR depressia[All Fields] OR depressiakh[All Fields] OR depressiami[All Fields] OR depressibilite[All Fields] OR depressibility[All Fields] OR depressible[All Fields] OR depressibles[All Fields] OR depressicapitatus[All Fields] OR depressice[All Fields] OR depressiceps[All Fields] OR depressiclypealisli[All Fields] OR depressicornis[All Fields] OR depressie[All Fields] OR depressie'[All Fields] OR depressiebehandeling[All Fields] OR depressief[All Fields] OR depressieherkenningsschaal[All Fields] OR depressieherkenningsschaal'[All Fields] OR depressiehypothese[All Fields] OR depressiei[All Fields] OR depressielijst[All Fields] OR depressies[All Fields] OR depressietoestanden[All Fields] OR depressietrials[All Fields] OR depressieve[All Fields] OR depressievragenlijst[All Fields] OR depressiewe[All Fields] OR depressif[All Fields] OR depressiferussp[All Fields] OR depressiform[All Fields] OR depressifrons[All Fields] OR depressifs[All Fields] OR depressigyra[All Fields] OR depressii[All Fields] OR depressiia[All Fields] OR depressiiakh[All Fields] OR depressiiami[All Fields] OR depressiion[All Fields] OR depressiiu[All Fields] OR depressiivisen[All Fields] OR depressiiviset[All Fields] OR depressiivisyys[All Fields] OR depressil6i[All Fields] OR depressin[All Fields] OR depressina[All Fields] OR depressine[All Fields] OR depressing[All Fields] OR depressing'[All Fields] OR depressingly[All Fields] OR depressinom[All Fields] OR depressins[All Fields] OR depressio[All Fields] OR depressioalttiuden[All Fields] OR depressiogene[All Fields] OR depressiogenic[All Fields] OR depressiok[All Fields] OR depressiokrol[All Fields] OR depressiolaake[All Fields] OR depressiolaakkeet[All Fields] OR depressiolaakkeiden[All Fields] OR depressiolaakkeilla[All Fields] OR depressiolytic[All Fields] OR depression[All Fields] OR depression'[All Fields] OR depression''[All Fields] OR depression's[All Fields] OR depression,[All Fields] OR depression1[All Fields] OR depression2[All Fields] OR depressiona[All Fields] OR depressional[All Fields] OR depressionars[All Fields] OR depressionbipolarclinic[All Fields] OR depressionby[All Fields] OR depressioncenter[All Fields] OR depressiondagger[All Fields] OR depressione[All Fields] OR depressionegravidanza[All Fields] OR depressionen[All Fields] OR depressionenegli[All Fields] OR depressionens[All Fields] OR depressioner[All Fields] OR depressionera[All Fields] OR depressioners[All Fields] OR depressioneur[All Fields] OR depressionevoking[All Fields] OR depressioni[All Fields] OR depressioniciclotimiche[All Fields] OR depressionless[All Fields] OR depressionlevels[All Fields] OR depressionlike[All Fields] OR depressionnogo[All Fields] OR depressionogenic[All Fields] OR depressionpsd[All Fields] OR depressions[All Fields] OR depressions'[All Fields] OR depressionsabgrenzung[All Fields] OR depressionsaequivalent[All Fields] OR depressionsapparate[All Fields] OR depressionsbedingten[All Fields] OR depressionsbeginns[All Fields] OR depressionsbegrebet[All Fields] OR depressionsbegriffes[All Fields] OR depressionsbehandling[All Fields] OR depressionsbehandlingen[All Fields] OR depressionsbehandlung[All Fields] OR depressionsbetinget[All Fields] OR depressionsdatabase[All Fields] OR depressionsdiagnose[All Fields] OR depressionsdiagnostik[All Fields] OR depressionsersatz[All Fields] OR depressionserschienungen[All Fields] OR depressionsfall[All Fields] OR depressionsform[All Fields] OR depressionsformen[All Fields] OR depressionsforschung[All Fields] OR depressionsforschungsabteilung[All Fields] OR depressionsforskningen[All Fields] OR depressionsgebietes[All Fields] OR depressionshaufigkeit[All Fields] OR depressionshilfe[All Fields] OR depressionsimmunitat[All Fields] OR depressionsinventar[All Fields] OR depressionsinventars[All Fields] OR depressionskonzept[All Fields] OR depressionsleitlinien[All Fields] OR depressionsmodellen[All Fields] OR depressionsmoduls[All Fields] OR depressionsneurose[All Fields] OR depressionspatienten[All Fields] OR depressionsquotienten[All Fields] OR depressionsrecidiv[All Fields] OR depressionsrisiko[All Fields] OR depressionsrisken[All Fields] OR depressionssation[All Fields] OR depressionsscreening[All Fields] OR depressionsscreenings[All Fields] OR depressionssjukdomar[All Fields] OR depressionsskala[All Fields] OR depressionsskalen[All Fields] OR depressionsskele[All Fields] OR depressionsspezifischer[All Fields] OR depressionsstation[All Fields] OR depressionsstationen[All Fields] OR depressionssygdom[All Fields] OR depressionssygdommen[All Fields] OR depressionssymptomatik[All Fields] OR depressionssymptome[All Fields] OR depressionssymptomen[All Fields] OR depressionssymptomer[All Fields] OR depressionssyndroms[All Fields] OR depressionstecken[All Fields] OR depressionstest[All Fields] OR depressionstests[All Fields] OR depressionstherapie[All Fields] OR depressionstiefe[All Fields] OR depressionstillstand[All Fields] OR depressionstilstande[All Fields] OR depressionstype[All Fields] OR depressionsverlauf[All Fields] OR depressionsvorgange[All Fields] OR depressionszentrum[All Fields] OR depressionszustaende[All Fields] OR depressionszustamden[All Fields] OR depressionszustand[All Fields] OR depressionszustande[All Fields] OR depressionszustanden[All Fields] OR depressiont1[All Fields] OR depressiont2[All Fields] OR depressionto[All Fields] OR depressioon[All Fields] OR depressiopotilaiden[All Fields] OR depressiosn[All Fields] OR depressiot[All Fields] OR depressiota[All Fields] OR depressiotausta[All Fields] OR depressiotilojen[All Fields] OR depressirostris[All Fields] OR depressirovannykh[All Fields] OR depressirovannym[All Fields] OR depressis[All Fields] OR depressissimum[All Fields] OR depressithorax[All Fields] OR depressiur[All Fields] OR depressiusculum[All Fields] OR depressiv[All Fields] OR depressiva[All Fields] OR depressivas[All Fields] OR depressive[All Fields] OR depressive'[All Fields] OR depressive's[All Fields] OR depressivedepressive[All Fields] OR depressivedisorders[All Fields] OR depressivelike[All Fields] OR depressively[All Fields] OR depressivem[All Fields] OR depressivemood[All Fields] OR depressivemood'[All Fields] OR depressiven[All Fields] OR depressiveness[All Fields] OR depressivenss[All Fields] OR depressiver[All Fields] OR depressives[All Fields] OR depressives'[All Fields] OR depressivesymptoms[All Fields] OR depressivi[All Fields] OR depressivitat[All Fields] OR depressivitats[All Fields] OR depressivitatsmessung[All Fields] OR depressivitatsskala[All Fields] OR depressivite[All Fields] OR depressiviteit[All Fields] OR depressivities[All Fields] OR depressivity[All Fields] OR depressivness[All Fields] OR depressivno[All Fields] OR depressivnoe[All Fields] OR depressivnogo[All Fields] OR depressivnoi[All Fields] OR depressivnom[All Fields] OR depressivnopodobnogo[All Fields] OR depressivnopodobnye[All Fields] OR depressivnye[All Fields] OR depressivnyi[All Fields] OR depressivnykh[All Fields] OR depressivnyky[All Fields] OR depressivnym[All Fields] OR depressivnymi[All Fields] OR depressivnyye[All Fields] OR depressivo[All Fields] OR depressivos[All Fields] OR depressivse[All Fields] OR depressivus[All Fields] OR depressivykh[All Fields] OR depresso[All Fields] OR depressoculus[All Fields] OR depressoes[All Fields] OR depressogene[All Fields] OR depressogenes[All Fields] OR depressogenic[All Fields] OR depressogenic'[All Fields] OR depressogennogo[All Fields] OR depressogennom[All Fields] OR depressogenous[All Fields] OR depressolytique[All Fields] OR depresson[All Fields] OR depressor[All Fields] OR depressor'[All Fields] OR depressor's[All Fields] OR depressorafferes[All Fields] OR depressoras[All Fields] OR depressore[All Fields] OR depressores[All Fields] OR depressori[All Fields] OR depressoric[All Fields] OR depressorisch[All Fields] OR depressorische[All Fields] OR depressorischen[All Fields] OR depressorischer[All Fields] OR depressormoi[All Fields] OR depressormymi[All Fields] OR depressornaia[All Fields] OR depressorno[All Fields] OR depressornoe[All Fields] OR depressornog[All Fields] OR depressornogo[All Fields] OR depressornoi[All Fields] OR depressornom[All Fields] OR depressornuiu[All Fields] OR depressornye[All Fields] OR depressornykh[All Fields] OR depressorotunda[All Fields] OR depressorreflex[All Fields] OR depressors[All Fields] OR depressory[All Fields] OR depressoside[All Fields] OR depressosides[All Fields] OR depressotetrix[All Fields] OR depressotypal[All Fields] OR depressotypic[All Fields] OR depressped[All Fields] OR depresss[All Fields] OR depresssion[All Fields] OR depresssive[All Fields] OR depresssor[All Fields] OR depressum[All Fields] OR depressure[All Fields] OR depressurisation[All Fields] OR depressurise[All Fields] OR depressurised[All Fields] OR depressurising[All Fields] OR depressurization[All Fields] OR depressurizations[All Fields] OR depressurize[All Fields] OR depressurized[All Fields] OR depressurizes[All Fields] OR depressurizing[All Fields] OR depressurs[All Fields] OR depressus[All Fields] OR depressvie[All Fields] OR depresszans[All Fields] OR depresszio[All Fields] OR depresszioban[All Fields] OR depressziok[All Fields] OR depressziora[All Fields] OR depresszios[All Fields] OR depressziot[All Fields] OR depresszioval[All Fields] OR depressziv[All Fields]) AND Meta-Analysis[ptyp] |
| #3 | (depress[All Fields] OR depressa[All Fields] OR depressability[All Fields] OR depressable[All Fields] OR depressan[All Fields] OR depressana[All Fields] OR depressant[All Fields] OR depressant'[All Fields] OR depressantami[All Fields] OR depressantov[All Fields] OR depressants[All Fields] OR depressants'[All Fields] OR depressao[All Fields] OR depressaria[All Fields] OR depressariidae[All Fields] OR depresse[All Fields] OR depressed[All Fields] OR depressed'[All Fields] OR depressed''[All Fields] OR depressedosteoblast[All Fields] OR depresseed[All Fields] OR depressei[All Fields] OR depresseion[All Fields] OR depressent[All Fields] OR depresser[All Fields] OR depresses[All Fields] OR depressesed[All Fields] OR depressess[All Fields] OR depresseur[All Fields] OR depresseurs[All Fields] OR depresseux[All Fields] OR depressi[All Fields] OR depressi'[All Fields] OR depressia[All Fields] OR depressiakh[All Fields] OR depressiami[All Fields] OR depressibilite[All Fields] OR depressibility[All Fields] OR depressible[All Fields] OR depressibles[All Fields] OR depressicapitatus[All Fields] OR depressice[All Fields] OR depressiceps[All Fields] OR depressiclypealisli[All Fields] OR depressicornis[All Fields] OR depressie[All Fields] OR depressie'[All Fields] OR depressiebehandeling[All Fields] OR depressief[All Fields] OR depressieherkenningsschaal[All Fields] OR depressieherkenningsschaal'[All Fields] OR depressiehypothese[All Fields] OR depressiei[All Fields] OR depressielijst[All Fields] OR depressies[All Fields] OR depressietoestanden[All Fields] OR depressietrials[All Fields] OR depressieve[All Fields] OR depressievragenlijst[All Fields] OR depressiewe[All Fields] OR depressif[All Fields] OR depressiferussp[All Fields] OR depressiform[All Fields] OR depressifrons[All Fields] OR depressifs[All Fields] OR depressigyra[All Fields] OR depressii[All Fields] OR depressiia[All Fields] OR depressiiakh[All Fields] OR depressiiami[All Fields] OR depressiion[All Fields] OR depressiiu[All Fields] OR depressiivisen[All Fields] OR depressiiviset[All Fields] OR depressiivisyys[All Fields] OR depressil6i[All Fields] OR depressin[All Fields] OR depressina[All Fields] OR depressine[All Fields] OR depressing[All Fields] OR depressing'[All Fields] OR depressingly[All Fields] OR depressinom[All Fields] OR depressins[All Fields] OR depressio[All Fields] OR depressioalttiuden[All Fields] OR depressiogene[All Fields] OR depressiogenic[All Fields] OR depressiok[All Fields] OR depressiokrol[All Fields] OR depressiolaake[All Fields] OR depressiolaakkeet[All Fields] OR depressiolaakkeiden[All Fields] OR depressiolaakkeilla[All Fields] OR depressiolytic[All Fields] OR depression[All Fields] OR depression'[All Fields] OR depression''[All Fields] OR depression's[All Fields] OR depression,[All Fields] OR depression1[All Fields] OR depression2[All Fields] OR depressiona[All Fields] OR depressional[All Fields] OR depressionars[All Fields] OR depressionbipolarclinic[All Fields] OR depressionby[All Fields] OR depressioncenter[All Fields] OR depressiondagger[All Fields] OR depressione[All Fields] OR depressionegravidanza[All Fields] OR depressionen[All Fields] OR depressionenegli[All Fields] OR depressionens[All Fields] OR depressioner[All Fields] OR depressionera[All Fields] OR depressioners[All Fields] OR depressioneur[All Fields] OR depressionevoking[All Fields] OR depressioni[All Fields] OR depressioniciclotimiche[All Fields] OR depressionless[All Fields] OR depressionlevels[All Fields] OR depressionlike[All Fields] OR depressionnogo[All Fields] OR depressionogenic[All Fields] OR depressionpsd[All Fields] OR depressions[All Fields] OR depressions'[All Fields] OR depressionsabgrenzung[All Fields] OR depressionsaequivalent[All Fields] OR depressionsapparate[All Fields] OR depressionsbedingten[All Fields] OR depressionsbeginns[All Fields] OR depressionsbegrebet[All Fields] OR depressionsbegriffes[All Fields] OR depressionsbehandling[All Fields] OR depressionsbehandlingen[All Fields] OR depressionsbehandlung[All Fields] OR depressionsbetinget[All Fields] OR depressionsdatabase[All Fields] OR depressionsdiagnose[All Fields] OR depressionsdiagnostik[All Fields] OR depressionsersatz[All Fields] OR depressionserschienungen[All Fields] OR depressionsfall[All Fields] OR depressionsform[All Fields] OR depressionsformen[All Fields] OR depressionsforschung[All Fields] OR depressionsforschungsabteilung[All Fields] OR depressionsforskningen[All Fields] OR depressionsgebietes[All Fields] OR depressionshaufigkeit[All Fields] OR depressionshilfe[All Fields] OR depressionsimmunitat[All Fields] OR depressionsinventar[All Fields] OR depressionsinventars[All Fields] OR depressionskonzept[All Fields] OR depressionsleitlinien[All Fields] OR depressionsmodellen[All Fields] OR depressionsmoduls[All Fields] OR depressionsneurose[All Fields] OR depressionspatienten[All Fields] OR depressionsquotienten[All Fields] OR depressionsrecidiv[All Fields] OR depressionsrisiko[All Fields] OR depressionsrisken[All Fields] OR depressionssation[All Fields] OR depressionsscreening[All Fields] OR depressionsscreenings[All Fields] OR depressionssjukdomar[All Fields] OR depressionsskala[All Fields] OR depressionsskalen[All Fields] OR depressionsskele[All Fields] OR depressionsspezifischer[All Fields] OR depressionsstation[All Fields] OR depressionsstationen[All Fields] OR depressionssygdom[All Fields] OR depressionssygdommen[All Fields] OR depressionssymptomatik[All Fields] OR depressionssymptome[All Fields] OR depressionssymptomen[All Fields] OR depressionssymptomer[All Fields] OR depressionssyndroms[All Fields] OR depressionstecken[All Fields] OR depressionstest[All Fields] OR depressionstests[All Fields] OR depressionstherapie[All Fields] OR depressionstiefe[All Fields] OR depressionstillstand[All Fields] OR depressionstilstande[All Fields] OR depressionstype[All Fields] OR depressionsverlauf[All Fields] OR depressionsvorgange[All Fields] OR depressionszentrum[All Fields] OR depressionszustaende[All Fields] OR depressionszustamden[All Fields] OR depressionszustand[All Fields] OR depressionszustande[All Fields] OR depressionszustanden[All Fields] OR depressiont1[All Fields] OR depressiont2[All Fields] OR depressionto[All Fields] OR depressioon[All Fields] OR depressiopotilaiden[All Fields] OR depressiosn[All Fields] OR depressiot[All Fields] OR depressiota[All Fields] OR depressiotausta[All Fields] OR depressiotilojen[All Fields] OR depressirostris[All Fields] OR depressirovannykh[All Fields] OR depressirovannym[All Fields] OR depressis[All Fields] OR depressissimum[All Fields] OR depressithorax[All Fields] OR depressiur[All Fields] OR depressiusculum[All Fields] OR depressiv[All Fields] OR depressiva[All Fields] OR depressivas[All Fields] OR depressive[All Fields] OR depressive'[All Fields] OR depressive's[All Fields] OR depressivedepressive[All Fields] OR depressivedisorders[All Fields] OR depressivelike[All Fields] OR depressively[All Fields] OR depressivem[All Fields] OR depressivemood[All Fields] OR depressivemood'[All Fields] OR depressiven[All Fields] OR depressiveness[All Fields] OR depressivenss[All Fields] OR depressiver[All Fields] OR depressives[All Fields] OR depressives'[All Fields] OR depressivesymptoms[All Fields] OR depressivi[All Fields] OR depressivitat[All Fields] OR depressivitats[All Fields] OR depressivitatsmessung[All Fields] OR depressivitatsskala[All Fields] OR depressivite[All Fields] OR depressiviteit[All Fields] OR depressivities[All Fields] OR depressivity[All Fields] OR depressivness[All Fields] OR depressivno[All Fields] OR depressivnoe[All Fields] OR depressivnogo[All Fields] OR depressivnoi[All Fields] OR depressivnom[All Fields] OR depressivnopodobnogo[All Fields] OR depressivnopodobnye[All Fields] OR depressivnye[All Fields] OR depressivnyi[All Fields] OR depressivnykh[All Fields] OR depressivnyky[All Fields] OR depressivnym[All Fields] OR depressivnymi[All Fields] OR depressivnyye[All Fields] OR depressivo[All Fields] OR depressivos[All Fields] OR depressivse[All Fields] OR depressivus[All Fields] OR depressivykh[All Fields] OR depresso[All Fields] OR depressoculus[All Fields] OR depressoes[All Fields] OR depressogene[All Fields] OR depressogenes[All Fields] OR depressogenic[All Fields] OR depressogenic'[All Fields] OR depressogennogo[All Fields] OR depressogennom[All Fields] OR depressogenous[All Fields] OR depressolytique[All Fields] OR depresson[All Fields] OR depressor[All Fields] OR depressor'[All Fields] OR depressor's[All Fields] OR depressorafferes[All Fields] OR depressoras[All Fields] OR depressore[All Fields] OR depressores[All Fields] OR depressori[All Fields] OR depressoric[All Fields] OR depressorisch[All Fields] OR depressorische[All Fields] OR depressorischen[All Fields] OR depressorischer[All Fields] OR depressormoi[All Fields] OR depressormymi[All Fields] OR depressornaia[All Fields] OR depressorno[All Fields] OR depressornoe[All Fields] OR depressornog[All Fields] OR depressornogo[All Fields] OR depressornoi[All Fields] OR depressornom[All Fields] OR depressornuiu[All Fields] OR depressornye[All Fields] OR depressornykh[All Fields] OR depressorotunda[All Fields] OR depressorreflex[All Fields] OR depressors[All Fields] OR depressory[All Fields] OR depressoside[All Fields] OR depressosides[All Fields] OR depressotetrix[All Fields] OR depressotypal[All Fields] OR depressotypic[All Fields] OR depressped[All Fields] OR depresss[All Fields] OR depresssion[All Fields] OR depresssive[All Fields] OR depresssor[All Fields] OR depressum[All Fields] OR depressure[All Fields] OR depressurisation[All Fields] OR depressurise[All Fields] OR depressurised[All Fields] OR depressurising[All Fields] OR depressurization[All Fields] OR depressurizations[All Fields] OR depressurize[All Fields] OR depressurized[All Fields] OR depressurizes[All Fields] OR depressurizing[All Fields] OR depressurs[All Fields] OR depressus[All Fields] OR depressvie[All Fields] OR depresszans[All Fields] OR depresszio[All Fields] OR depresszioban[All Fields] OR depressziok[All Fields] OR depressziora[All Fields] OR depresszios[All Fields] OR depressziot[All Fields] OR depresszioval[All Fields] OR depressziv[All Fields]) AND (meta analyis[All Fields] OR meta analyisis[All Fields] OR meta analysable[All Fields] OR meta analysas[All Fields] OR meta analyse[All Fields] OR meta analysed[All Fields] OR meta analysei[All Fields] OR meta analysen[All Fields] OR meta analyser[All Fields] OR meta analysers[All Fields] OR meta analyses[All Fields] OR meta analysescohort[All Fields] OR meta analysespublication[All Fields] OR meta analysestype[All Fields] OR meta analysi[All Fields] OR meta analysia[All Fields] OR meta analysic[All Fields] OR meta analysing[All Fields] OR meta analysis[All Fields] OR meta analysis's[All Fields] OR meta analysis,[All Fields] OR meta analysis2011[All Fields] OR meta analysisdagger[All Fields] OR meta analysises[All Fields] OR meta analysisevaluating[All Fields] OR meta analysisif[All Fields] OR meta analysisintroduction[All Fields] OR meta analysisjr[All Fields] OR meta analysismoderate[All Fields] OR meta analysisof[All Fields] OR meta analysisv[All Fields] OR meta analysized[All Fields] OR meta analyst[All Fields] OR meta analysticians[All Fields] OR meta analysts[All Fields] OR meta analysys[All Fields] OR meta analytic[All Fields] OR meta analytical[All Fields] OR meta analytically[All Fields] OR meta analytics[All Fields] OR meta analytischer[All Fields] OR meta analyysit[All Fields] OR meta analyza[All Fields] OR meta analyzable[All Fields] OR meta analyze[All Fields] OR meta analyzed[All Fields] OR meta analyzes[All Fields] OR meta analyzing[All Fields]) |
| #4 | (#1 OR #2 OR #3) AND ("2014/01/01"[PDAT] : "2015/12/31"[PDAT]) |
